# Socioeconomic determinants of cancer screening adherence among cancer survivors: analysis from the 2020 Behavioral Risk Factor Surveillance System

**DOI:** 10.1093/jncics/pkae127

**Published:** 2024-12-19

**Authors:** Edoardo Beatrici, Zhiyu Qian, Dejan K Filipas, Benjamin V Stone, Filippo Dagnino, Muhieddine Labban, Stuart R Lipsitz, Giovanni Lughezzani, Nicolò M Buffi, Alexander P Cole, Quoc-Dien Trinh

**Affiliations:** Department of Urology and Center for Surgery and Public Health, Brigham and Women’s Hospital, Harvard Medical School, Boston, MA 02115, United States; Department of Urology, Humanitas Research Hospital—IRCCS, Milan 20121, Italy; Department of Urology and Center for Surgery and Public Health, Brigham and Women’s Hospital, Harvard Medical School, Boston, MA 02115, United States; Department of Urology and Center for Surgery and Public Health, Brigham and Women’s Hospital, Harvard Medical School, Boston, MA 02115, United States; Department of Urology, University Medical Center Hamburg-Eppendorf, Hamburg 20251, Germany; Department of Urology and Center for Surgery and Public Health, Brigham and Women’s Hospital, Harvard Medical School, Boston, MA 02115, United States; Department of Urology and Center for Surgery and Public Health, Brigham and Women’s Hospital, Harvard Medical School, Boston, MA 02115, United States; Department of Urology, Humanitas Research Hospital—IRCCS, Milan 20121, Italy; Department of Urology and Center for Surgery and Public Health, Brigham and Women’s Hospital, Harvard Medical School, Boston, MA 02115, United States; Department of Urology and Center for Surgery and Public Health, Brigham and Women’s Hospital, Harvard Medical School, Boston, MA 02115, United States; Department of Medicine, Brigham and Women’s Hospital, Harvard Medical School, Boston, MA 20251, United States; Department of Urology, Humanitas Research Hospital—IRCCS, Milan 20121, Italy; Department of Urology, Humanitas Research Hospital—IRCCS, Milan 20121, Italy; Department of Urology and Center for Surgery and Public Health, Brigham and Women’s Hospital, Harvard Medical School, Boston, MA 02115, United States; Department of Urology and Center for Surgery and Public Health, Brigham and Women’s Hospital, Harvard Medical School, Boston, MA 02115, United States

## Abstract

**Background:**

Factors associated with cancer survivors’ preventive health behaviors are understudied. We hypothesized that socioeconomic and health-care access factors may be associated with adherence to recommended cancer screenings.

**Methods:**

We conducted a cross-sectional analysis using the 2020 Behavioral Risk Factor Surveillance System. Cancer survivors eligible for United States Preventive Services Task Force–recommended breast, cervical, prostate, and colorectal screenings were included. Multivariable logistic regression models were used to identify socioeconomic factors significantly associated with screening adherence.

**Results:**

Overall, 64 958 (weighted national estimate = 29 066 143) cancer survivors were included. Adherence rates varied across cancer types: 80.9% for breast, 88.9% for cervical, 54.1% for prostate, and 84.7% for colorectal cancer. Key predictors of low adherence included lower income (breast: adjusted odds ratio [aOR] = 0.56, 95% confidence interval [CI] = 0.43 to 0.74; cervical: aOR = 0.38, 95% CI = 0.24 to 0.59; prostate: aOR = 0.36, 95% CI = 0.24 to 0.52; colorectal: aOR = 0.74, 95% CI = 0.57 to 0.96), lack of health-care coverage for colorectal cancer (aOR = 0.51, 95% CI = 0.36 to 0.73), time since last checkup between 1 and 2 years prior for breast (aOR = 0.58, 95% CI = 0.45 to 0.75), prostate (aOR = 0.66, 95% CI = 0.47 to 0.91), and colorectal (aOR = 0.69, 95% CI = 0.56 to 0.86) cancer, and no health-care provider for breast (aOR = 0.68, 95% CI = 0.47 to 0.98), prostate (aOR = 0.45, 95% CI = 0.31 to 0.65), and colorectal (aOR = 0.51, 95% CI = 0.40 to 0.66) cancer.

**Conclusion:**

Cancer survivors’ adherence to screening is associated with factors including lack of health-care coverage, lower income, time since the last exam, and having a personal provider. Targeted interventions accounting for such factors may help mitigate these disparities.

## Introduction

With the incidence of US cancers rising to about 2 million new cases in 2023, significant public health concerns have emerged.^1^^,^^2^ In 1986, the National Coalition for Cancer Survivorship replaced the term “cancer victim” with “cancer survivor”^3^ to better address the diverse challenges of this growing population^4^ and to shift the perception of these patients from mere health-care recipients to empowered individuals making informed decisions.^5^ Factors such as sociodemographic attributes, geographic locations, and income levels determine economic barriers that are associated with cancer care quality and patient outcomes.^6^ Limited data on catastrophic health-care expenses among specific cancer survivor groups highlight these challenges,^7^ posing a potential obstacle to subsequent care.

Previous studies indicate that among cancer survivors diagnosed with a second malignancy, 31% are breast cancers, 4% are cervical cancers, 9% are prostate cancers, and 12% are colorectal cancers.^8^^,^^9^ Given this elevated risk, analyzing adherence to screening guidelines for prevalent cancers—breast, cervical, prostate, and colorectal—is crucial in this vulnerable population.^10^ This study uses data from the Behavioral Risk Factor Surveillance System (BRFSS) to examine socioeconomic factors associated with adherence to screening guidelines among cancer survivors for the 4 most common cancers, aiming to inform strategies for preventing second malignancies.

## Methods

### Data source

The BRFSS, overseen by the Centers for Disease Control and Prevention, conducts yearly telephone interviews nationwide, making it the largest ongoing health survey globally. It gathers data on health risk activities, long-term conditions, and preventive health-care uptake. To ensure representation and reduce biases, BRFSS uses iterative proportional fitting and includes cell phone responses.^11^ This study used 2020 survey data, known for preventive health questions in even years, with a weighted national response rate of 47.9%.

### Study population

The cohort included patients who answered “Yes” to “Has a doctor ever told you had skin cancer?” (CHCSCNCR) or “Has a doctor ever told you had any other types of cancer?” (CHCOCNCR), excluding those with missing responses. Stratification by cancer type (CNCRTYP1) grouped them into breast, cervical, prostate, colorectal, and other cancers, excluding those with prior diagnoses of the cancers under study. For a refined sensitivity analysis, individuals with a history of skin cancer were excluded, focusing on those with “any other types of cancer.”

### Main outcome variable

Primary outcomes measured adherence to the United States Preventive Services Task Force (USPSTF) screening guidelines for breast, cervical, prostate, and colorectal cancers using BRFSS 2020 data. Participants with prior diagnoses of the respective cancer, or those answering “Don’t know/not sure” or “Refused” were excluded. Entries with unspecified cancers were included, ensuring a focused assessment of adherence among cancer survivors.

Women aged 50-74 were considered adherent to breast cancer screening if they had a mammogram within the past 2 years, in accordance with USPSTF guidelines; otherwise, they were nonadherent. For women aged 40-49, adherence was noted if a mammogram was done in the last 2 years, but nonadherence was not assigned due to USPSTF’s recommendation for individualized screening in this group.

Cervical cancer screening adherence was defined as a Pap test within 3 years for women aged 21-29, and either a Pap test within 3 years or a human papilloma virus test within 5 years for women aged 30-65. Nonadherence was if the last Pap test was 4 or more years ago for ages 21-29, or if neither test was done for ages 30-65. Women with a history of hysterectomy were excluded.

Without specific USPSTF recommendations for prostate-specific antigen (PSA) testing frequency, we used a biennial timeframe to assess adherence among men aged 55-69, following previous research as a practical and empirical proxy,^12^ aligning with USPSTF’s emphasis on individualized decision-making. Nonadherence was defined as no PSA test within this biennial window.

For colorectal cancer screening, adherence for individuals aged 50-75 was defined as completing 1 of these tests within the recommended timeframe: colonoscopy (10 years), sigmoidoscopy (5 years), fecal blood test (1 year), stool DNA test (3 years), or virtual colonoscopy (5 years). Nonadherence was defined as missing all tests beyond these intervals.

### Main predictor variables

To assess the socioeconomic impact of a prior cancer diagnosis on screening adherence, we used annual household income, health-care coverage, cost-related access barriers in the previous 12 months, time since the last checkup, and the presence of a personal doctor as primary predictors of cancer screening adherence.

Income was categorized into 4 levels: below $25 000, between $25 000 and $49 999, between $50 000 and $75 000, and above $75 000.^13^ Health-care coverage (any kind of health-care coverage, including health insurance, prepaid plans such as HMOs, or government plans such as Medicare or Indian Health Service), cost-related medical access barriers in the previous 12 months, and the presence of a personal doctor were classified as yes/no variables. Finally, the time since the last checkup was grouped into 4 categories: less than 12 months, 1 year but less than 2 years, 2 years but less than 5 years, and 5 years or more. Responses of “Don’t know/Not sure” or missing income data were categorized as “Don’t know/Not sure/Missing.”

### Covariates

Covariates included employment status categorized as employed, unemployed, not currently in the workforce, unable to work, or refused/missing; education level categorized as college graduate, some college or technical school, high school or graduated high school, never attended school or only elementary, refused; rural/urban status; marital status categorized as never married/unmarried couple, married, previously married, refused; age as a continuous variable; race/ethnicity categorized as White non-Hispanic, Black non-Hispanic, American Indian or Alaskan Native, Hispanic, American Indian or Alaskan Native, Other races. Biological sex (or sex at birth) was included only in the colorectal cancer analysis.

### Statistical analysis

The analysis used BRFSS weighting (_LLCPWT) to generalize sample findings, with stratification (_STSTR) and clustering (_PSU) defined. Descriptive statistics included frequencies and percentages for categorical variables and medians with interquartile ranges for continuous variables. Multivariable logistic regression models were constructed using predictors found to be statistically significant in univariable analyses. Multivariable models with complex samples estimated adjusted odds ratios (aORs) and 95% confidence intervals (CIs) for predictors of screening adherence. Statistical significance was set at a *P* value less than .05 (2-tailed). Stata software, version 17.0, was used for all analyses.

## Results

Among the study population, 64 680 participants were identified as cancer survivors, indicating a prior diagnosis of skin cancer, non-skin cancer, or both. This corresponded to a weighted national estimate (WNE) of 29 066 143 individuals. Overall, 28 060 or 43.4% (WNE = 12 271 347 or 42.2%) individuals had a documented history of skin cancer, 28 378 or 43.9% (WNE = 13 398 346 or 46.1%) reported diagnoses of non-skin cancers, and 8242 or 12.8% (WNE = 3 395 450 or 11.7%) indicated a history of both cancer categories. Baseline characteristics categorized by compliance with USPSTF guidelines for breast, cervical, prostate, and colorectal cancer screenings are reported in Table 1.

**Table 1.** Baseline characteristics of individuals categorized by compliance with United States Preventive Services Task Force guidelines for breast, cervical, prostate, and colorectal cancer screenings.

	Breast cancer screening			Cervical cancer screening			Prostate cancer screening			Colorectal cancer screening		
	Nonadherent no. (%)	Adherent no. (%)	*P*	Nonadherent no. (%)	Adherent no. (%)	*P*	Nonadherent no. (%)	Adherent no. (%)	*P*	Nonadherent no. (%)	Adherent no. (%)	*P*
	**N** ^a^ **= 1** **448** **616**	**N** ^a^ **= 6** **540** **335**		**N** ^a^ **= 470** **807**	**N** ^a^ **= 4** **212** **574**		**N** ^a^ **= 1** **721** **608**	**N** ^a^ **= 2** **149** **758**		**N** ^a^ **= 2** **684** **253**	**N** ^a^ **= 13** **351** **697**	
Annual household income			<.001			<.001			<.001			<.001
>$75 000 (base)	326 295 (22.5)	2 034 338 (31.1)		104 039 (22.1)	1 720 306 (40.8)		589 657 (34.3)	1 025 340 (47.7)		823 438 (30.6)	4 650 304 (37.7)	
$50 000-$75 000	173 405 (12.0)	924 234 (14.1)		52 027 (11.1)	467 722 (11.1)		207 596 (12.1)	304 023 (14.1)		328 226 (12.2)	1 919 403 (15.5)	
$25 000-$49 999	300 380 (20.7)	1 296 982 (19.8)		93 937 (20.0)	709 401 (16.8)		268 576 (15.6)	323 057 (15.0)		475 466 (17.7)	2 495 989 (20.2)	
<$25 000	384 154 (26.5)	1 032 283 (15.8)		141 797 (30.1)	673 054 (16.0)		426 591 (24.8)	225 662 (10.5)		630 251 (23.4)	2 057 947 (16.7)	
Don’t know/Not sure/Missing	264 382 (18.3)	1 252 498 (19.2)		79 007 (16.8)	642 091 (15.2)		226 187 (13.1)	271 675 (12.7)		426 873 (15.0)	2 228 053 (18.9)	
Health-care coverage			<.001			<.001			<.001			<.001
Yes (base)	1 338 894 (92.4)	6 365 310 (97.3)		420 627 (89.3)	3 961 633 (94.0)		1 607 683 (93.4)	2 102 031 (97.8)		2 437 677 (90.8)	13 043 720 (97.7)	
No	106 003 (7.3)	161 069 (2.5)		49 657 (10.6)	246 036 (5.8)		111 685 (6.5)	46 043 (2.1)		241 558 (9.0)	280 548 (2.1)	
Don’t know/Not sure/Missing	3719 (0.3)	13 955 (0.2)		523 (0.1)	4905 (0.1)		2240 (0.1)	1682 (0.1)		5017 (0.2)	27 428 (0.2)	
Cost-related medical access barrier in the previous 12 months			<.001			<.001			<.001			<.001
No (base)	1 229 446 (84.9)	6 101 341 (93.3)		392 351 (83.3)	3 697 027 (87.8)		1 556 638 (90.4)	2 058 496 (95.8)		2 341 903 (87.2)	12 566 852 (94.1)	
Yes	217 713 (15.0)	433 441 (6.6)		77 935 (16.6)	510 422 (12.1)		159 668 (9.3)	90 895 (4.2)		338 289 (12.6)	773 054 (5.8)	
Don’t know/Not sure/Missing	1457 (0.1)	5553 (0.1)		522 (0.1)	5124 (0.1)		5301 (0.3)	366 (0.0)		4060 (0.2)	11 791 (0.1)	
Time elapsed from last checkup visit			<.001			<.001			<.001			<.001
Less than 12 months (base)	1 108 626 (76.5)	5 986 375 (91.5)		333 738 (70.9)	3 592 776 (85.3)		1 357 765 (79.0)	1 961 747 (91.3)		2 060 375 (76.7)	12 134 813 (90.9)	
1 year but less than 2	142 626 (9.9)	421 637 (6.5)		47 379 (10.1)	417 336 (9.9)		169 912 (9.9)	163 196 (7.6)		256 445 (9.6)	892 807 (6.7)	
2 years but less than 5 years	95 058 (6.6)	56 659 (0.9)		32 102 (6.8)	123 405 (2.9)		112 456 (6.5)	14 369 (0.7)		163 705 (6.1)	184 416 (1.4)	
5 years or more	82 133 (5.7)	31 167 (0.5)		49 793 (10.6)	55 754 (1.3)		72 076 (4.2)	6372 (0.3)		163 571 (6.1)	82 107 (0.6)	
Never	3034 (0.2)	6460 (0.1)		747 (0.2)	3656 (0.1)		2755 (0.2)	254 (0.1)		8263 (0.3)	9182 (0.1)	
Don’t know/Not sure/Missing	17 140 (1.2)	38 036 (0.6)		7048 (1.5)	19 648 (0.5)		5644 (0.3)	3817 (0.2)		31 894 (1.2)	48 371 (0.4)	
Personal doctor or health-care provider			<.001			<.001			<.001			<.001
Yes (base)	1 284 383 (88.7)	6 252 585 (95.6)		391 025 (83.1)	3 802 089 (90.3)		1 528 947 (88.8)	2 068 447 (96.2)		2 294 895 (85.4)	12 778 422 (95.7)	
No	159 667 (11.0)	273 583 (4.2)		79 193 (16.8)	399 316 (9.5)		185 212 (10.8)	68 169 (3.2)		383 059 (14.2)	529 736 (4.0)	
Don’t know/Not sure/Refused	4566 (0.3)	14 167 (0.2)		589 (0.1)	11 168 (0.3)		7448 (0.4)	13 142 (0.6)		6299 (0.2)	43 539 (0.3)	
Employment status			<.001			<.001			<.001			<.001
Employed (base)	464 747 (32.1)	2 101 191 (32.1)		198 086 (42.1)	2 254 577 (53.5)		792 357 (46.0)	1 027 481 (47.8)		1.085 762 (40.5)	4 539 955 (34.0)	
Unemployed	123 149 (8.5)	366 242 (5.6)		35 754 (7.6)	407 410 (9.7)		112 636 (6.5)	70 009 (3.3)		203 885 (7.6)	587 946 (4.4)	
Not currently in the workforce	586 557 (40.5)	3 315 986 (50.7)		130 938 (27.8)	1 069 058 (25.4)		533 850 (31.0)	847 702 (39.4)		1 009 028 (37.6)	6 809 986 (51.0)	
Unable to work	265 425 (18.3)	722 862 (11.1)		103 657 (22.0)	462 898 (11.0)		273 325 (15.9)	201 001 (9.34)		358 493 (13.4)	1 360 047 (10.2)	
Refused/Missing	8.738 (0.6)	34 054 (0.5)		2 371 (0.5)	18 630 (0.4)		9 440 (0.6)	3 564 (0.2)		27 085 (1.0)	53 762 (0.4)	
Educational level			<.001			<.001			<.001			<.001
College graduate (base)	393 086 (27.1)	2 190 941 (33.5)		136 613 (29.0)	1 693 138 (40.2)		987 047 (57.4)	1 478 243 (68.7)		762 310 (28.4)	4 663 764 (34.9)	
Some college or technical school	541 494 (37.4)	2 158 468 (33)		146 263 (31.1)	1 294 508 (30.7)		662 437 (38.5)	630 090 (29.3)		864 775 (32.2)	4 317 202 (32.3)	
High school or graduated high school	480 945 (33.2)	2 017 233 (30.8)		178 053 (37.8)	1 131 454 (26.9)		66 569 (3.9)	13 566 (0.6)		931 881 (34.7)	4 049 857 (30.3)	
Never attended school or only elementary	29 841 (2.1)	162 394 (2.5)		9094 (1.9)	83 400 (2.0)		5555 (0.3)	27 861 (1.3)		119 368 (4.4)	272 396 (2.0)	
Refused	3249 (0.2)	11 299 (0.2)		784 (0.2)	10 073 (0.2)		0 (0)	0 (0)		5918 (0.2)	48 479 (0.4)	
Urban/Rural status			<.001			.14			.08			<.001
Urban (base)	1 305 874 (90.2)	6 029 313 (92.2)		428 769 (91.1)	3 946 528 (93.7)		1 563 900 (90.9)	1 968 021 (91.5)		2 433 593 (90.7)	12 206 175 (92.0)	
Rural	137 084 (9.5)	459 743 (7.0)		39 533 (8.4)	234 546 (5.6)		154 005 (8.9)	168 808 (7.9)		235 931 (8.8)	1 053 108 (7.9)	
Missing	5659 (0.3)	51 280 (0.8)		2505 (0.5)	31 500 (0.8)		3703 (0.2)	12 929 (0.6)		14 729 (0.6)	14 400 (0.1)	
Marital status			<.001			<.001			<.001			<.001
Never Married/Unmarried couple (base)	135 767 (9.4)	496 136 (7.6)		87 519 (18.6)	636 116 (15.1)		205 204 (11.9)	149 240 (6.9)		276 651 (10.3)	1 006 298 (7.5)	
Married	785 170 (54.2)	4 054 004 (62.0)		253 746 (53.9)	2 623 772 (62.3)		1 101 817 (64.0)	1 643 822 (76.3)		1 565 454 (58.3)	8 844 123 (66.2)	
Previously married	515 382 (35.6)	1 942 127 (29.7)		128 066 (27.2)	911 570 (21.6)		406 750 (23.6)	352 578 (16.4)		825 627 (30.7)	3 421 198 (25.6)	
Refused	12 297 (0.9)	48 067 (0.7)		1476 (0.3)	41 116 (1.0)		7836 (0.5)	4117 (0.2)		16 522 (0.6)	80 078 (0.6)	
Age at survey	63.6	64.7	<.001	54.9	55.0	.37	62.8	64.1	<.001	60.0	63.8	<.001
Race/Ethnicity			<.001			.09			.01			<.001
White non-Hispanic (base)	1 231 086 (85.0)	5 347 662 (81.8)		349 256 (74.2)	3 255 480 (77.3)		1 448 433 (84.2)	1 815 819 (84.5)		2 204 591 (82.1)	11 142 272 (83.5)	
Black non-Hispanic	42 076 (2.9)	327 769 (5.0)		19 540 (4.2)	209 219 (5.0)		83 746 (4.9)	131 394 (6.1)		113 802 (4.2)	821 600 (6.2)	
American Indian or Alaskan Native	13 961 (1.0)	57 681 (0.9)		3589 (0.8)	40 388 (1.0)		15 899 (0.9)	39 540 (1.8)		27 708 (1.0)	123 049 (0.9)	
Hispanic	102 292 (7.1)	546 397 (8.4)		62 656 (13.3)	513 507 (12.2)		96 731 (5.6)	92 494 (4.3)		234 606 (8.7)	748 128 (5.6)	
Asian American, Native Hawaiian, and Pacific Islander	10 176 (0.7)	103 768 (1.6)		22 190 (4.7)	54 113 (1.3)		18 676 (1.1)	18 779 (0.9)		36 839 (1.4)	152 577 (1.1)	
Other races	22 364 (1.5)	78 174 (1.2)		8524 (1.8)	70 110 (1.7)		25 279 (1.5)	19 297 (0.9)		37 383 (1.4)	156 517 (1.2)	
Missing	26 611 (1.8)	78 884 (1.2)		5052 (1.1)	69 757 (1.7)		52 844 (3.1)	12 435 (0.6)		29 324 (1.1)	207 554 (1.6)	
Gender												<.001
Male	− ^a^	–	–	–	–	–	–	–	–	1 088 737 (8.6)	6 216 376 (46.6)	
Female	–	–	–	–	–	–	–	–	–	11 595 516 (91.4)	7 135 321 (53.4)	

a Survey-weighted estimates.

### Adherence to USPSTF guidelines among cancer survivors

The analysis determined eligibility for breast cancer screening for 18 073 women (WNE = 7 988 951). Adherence to USPSTF guidelines was observed in 14 614 or 80.9% cases (WNE = 6 540 335 or 81.9%).

For cervical cancer, 8481 women were identified as eligible for screening (WNE = 4 683 381). Adherence to USPSTF guidelines was reported in 7481 or 88.9% cases (WNE = 4 212 574 or 89.9%).

Regarding prostate cancer, 7979 eligible men were identified as eligible for screening (WNE = 3 871 366). Adherence was reported in 4319 or 54.1% cases (WNE = 2 149 758 or 55.5%).

Last, a total of 35 813 individuals were eligible for colorectal cancer screening (WNE = 16 035 950). Adherence to USPSTF guidelines was found in 30 341 or 84.7% cases (WNE = 13 351 697 or 83.3%).

### Socioeconomic characteristics variation by adherence to cancer screening guidelines

Individuals adherent to cancer screening had a higher prevalence of reporting an annual household income of less than $75 000 (breast: 31.1% vs 22.5%, cervical: 40.8% vs 22.1%, prostate: 47.7% vs 34.3%, colorectal: 37.7% vs 30.6%; all *P* < .001). They also had a higher prevalence of health-care coverage (breast: 97.3% vs 92.4%, cervical: 94.0% vs 89.3%, prostate: 97.8% vs 93.4%, colorectal: 97.7% vs 90.8%; all *P* < .001), did not face cost-related medical access barriers in the past year (breast: 93.3% vs 84.9%, cervical: 87.8% vs 83.3%, prostate: 95.8% vs 90.4%, colorectal: 94.1% vs 87.2%; all *P* < .001), had a personal doctor (breast: 95.6% vs 88.7%, cervical: 90.3% vs 83.1%, prostate: 96.2% vs 88.8%, colorectal: 95.7% vs 85.4%; all *P* < .001), and had a checkup visit within the last year (breast: 91.5% vs 76.5%, cervical: 85.3% vs 70.9%, prostate: 91.3% vs 79.0%, colorectal: 90.9% vs 76.7%). For complete statistical details and additional data, refer to Table 1.

Table S1 reports the USPSTF-concordant screening adherence rates breast, cervical, prostate, and colorectal cancers derived from the sensitivity analysis, with individuals reporting solely a history of skin cancer being excluded from this subset. Adherence rates were consistent with those reported in the main analysis for each cancer type.

### Predictors of adherence to USPSTF guidelines among cancer survivors

Table 2 provides a detailed description of the adjusted odds ratios for each predictor in relation to adherence across the 4 distinct cancer screenings.

**Table 2.** Multivariable logistic regression models analyzing predictors of compliance to United States Preventive Services Task Force guidelines for breast, cervical, prostate, and colorectal cancer screenings.

	Breast cancer screening			Cervical cancer screening			Prostate cancer screening			Colorectal cancer screening		
	Overall = 7 988 951			Overall = 4 683 381			Overall = 3 871 366			Overall = 16 035 950		
	aOR	95% CIs	*P*	aOR	95% CIs	*P*	aOR	95% CIs	*P*	aOR	95% CIs	*P*
Annual household income												
>$75 000 (base)	1.00			1.00			1.00			1.00		
$50 000-$75 000	0.88	0.67 to 1.26	.38	0.66	0.40 to 1.07	.93	0.85	0.64 to 1.14	.29	0.94	0.74 to 1.21	.65
$25 000-$49 999	0.74	0.53 to 1.04	.08	0.57	0.30 to 1.08	.09	0.68	0.50 to 0.94	.02	0.99	0.77 to 1.26	.91
<$25 000	0.56	0.43 to 0.74	<.001	0.38	0.24 to 0.59	<.001	0.36	0.24 to 0.52	<.001	0.74	0.57 to 0.96	.01
Don’t know/Not sure/Missing	0.79	0.60 to 1.03	.08	0.56	0.37 to 0.85	.01	0.61	0.45 to 0.81	.001	0.87	0.66 to 1.15	.32
Health-care coverage												
Yes (base)	1.00			1.00			1.00			1.00		
No	0.71	0.41 to 1.25	.23	0.79	0.46 to 1.38	.41	0.81	0.41 to 1.61	.55	0.51	0.36 to 0.73	<.001
Cost-related medical access barrier in the previous 12 months												
No (base)	1.00			1.00			1.00			1.00		
Yes	0.62	0.46 to 0.82	<.001	1.18	0.76 to 1.85	.46	0.78	0.52 to 1.18	.24	0.86	0.67 to 1.11	.25
Time elapsed from last checkup visit												
Less than 12 months (base)	1.00			1.00			1.00			1.00		
1 year but less than 2	0.58	0.45 to 0.75	<.001	0.71	0.47 to 1.06	.10	0.66	0.47 to 0.91	.01	0.69	0.56 to 0.86	.001
2 years but less than 5 years	0.14	0.09 to 0.22	<.001	0.26	0.15 to 0.46	<.001	0.12	0.05 to 0.25	<.001	0.27	0.18 to 0.40	<.001
5 years or more	0.10	0.06 to 0.19	<.001	0.09	0.05 to 0.17	<.001	0.10	0.04 to 0.26	<.001	0.13	0.08 to 0.20	<.001
Don’t know/Not sure/Missing	0.64	0.23 to 1.75	.39	0.74	0.13 to 4.07	.73	0.09	0.02 to 0.53	.01	0.28	0.13 to 0.62	<.001
Personal doctor or health-care provider												
Yes (base)	1.00			1.00			1.00			1.00		
No	0.68	0.47 to 0.98	.04	1.04	0.65 to 1.69	.86	0.45	0.31 to 0.65	<.001	0.51	0.40 to 0.66	<.001
Employment status												
Employed (base)	1.00			1.00			1.00			1.00		
Unemployed	0.77	0.34 to 1.74	.52	1.46	0.83 to 2.57	.18	0.81	0.46 to 1.44	.47	1.05	0.74 to 1.49	.79
Not currently in the workforce	1.08	0.86 to 1.35	.50	0.92	0.62 to 1.37	.68	1.23	0.98 to 1.54	.08	1.10	0.92 to 1.33	.30
Unable to work	0.71	0.53 to 0.93	.01	0.68	0.42 to 1.10	.12	1.07	0.74 to 1.53	.73	1.14	0.87 to 1.49	.33
Educational level												
College graduate (base)	1.00			1.00			1.00			1.00		
Some college or technical school	0.79	0.65 to 0.95	.01	0.73	0.53 to 1.01	.06	0.76	0.60 to 0.96	.02	0.83	0.69 to 1.00	.05
High school or graduated high school	0.87	0.69 to 1.01	.23	0.61	0.41 to 0.91	.02	0.66	0.51 to 0.85	.01	0.80	0.64 to 0.99	.04
Never attended school or only elementary	1.59	0.57 to 4.40	.38	1.31	0.48 to 3.64	.59	0.14	0.06 to 0.31	<.001	0.53	0.30 to 0.94	.03
Urban/Rural status												
Urban (base)	1.00			1.00			1.00			1.00		
Rural	0.79	0.63 to 0.98	.03	0.83	0.58 to 1.18	.31	1.09	0.83 to 1.44	.53	0.92	0.77 to 1.11	.39
Marital status												
Never married/Unmarried couple (base)	1.00			1.00			1.00			1.00		
Married	1.11	0.86 to 1.43	.43	1.17	0.81 to 1.70	.40	1.44	1.01 to 2.06	.05	1.31	1.03 to 1.67	.03
Previously married	0.90	0.69 to 1.16	.41	1.17	0.78 to 1.77	.46	1.12	0.77 to 1.63	.57	1.03	0.79 to 1.34	.82
Age at survey	1.01	0.99 to 1.04	.12	0.98	0.97 to 0.99	.01	1.07	1.04 to 1.10	<.001	1.05	1.04 to 1.07	<.001
Race/Ethnicity												
White non-Hispanic (base)	1.00			1.00			1.00			1.00		
Black non-Hispanic	2.09	1.46 to 3.00	<.001	1.58	0.78 to 3.20	.20	1.46	0.88 to 2.43	.14	1.56	1.07 to 2.27	.02
American Indian or Alaskan Native	1.22	0.72 to 2.07	.46	1.84	0.67 to 5.04	.24	3.39	0.74 to 15.33	.11	1.18	0.60 to 2.30	.64
Hispanic	1.58	0.61 to 4.10	.34	0.99	0.34 to 2.85	.99	1.43	0.81 to 2.51	.22	0.99	0.62 to 1.59	.96
Asian American, Native Hawaiian, and Pacific Islander	2.15	0.93 to 4.97	.07	0.22	0.07 to 0.68	.01	0.89	0.29 to 2.75	.84	0.66	0.28 to 1.52	.33
Other races	0.98	0.64 to 1.50	.93	2.16	0.54 to 8.60	.28	0.65	0.34 to 1.25	.20	1.01	0.71 to 1.44	.94
Gender												
Male	− ^a^	–	–	–	–	–	–	–	–	1.00		
Female	–	–	–	–	–	–	–	–	–	0.81	0.69 to 0.94	.01

Regarding the annual household income, compared with individuals with income higher than $75 000, those with lower income reported lower odds of USPSTF concordant screening adherence for all the cancers under examination. Specifically, individuals with income lower than $25 000 reported lower odds of breast (aOR = 0.56, 95% CI = 0.43 to 0.74; *P* < .001), cervical (aOR = 0.38, 95% CI = 0.24 to 0.59; *P* < .001), prostate (aOR = 0.36, 95% CI = 0.24 to 0.52; *P* < .001), and colorectal (aOR = 0.74, 95% CI = 0.57 to 0.96; *P* = .01) cancer screenings.

Compared with individuals with availability of a health-care coverage, those with no health-care coverage reported lower odds of adherence to colorectal cancer screening guidelines (aOR = 0.51, 95% CI = 0.36 to 0.73; *P* < .001), whereas no significant differences were found for the other cancer screenings.

Cost-related medical access barriers in the previous 12 months were significant predictors of nonadherence to breast (aOR = 0.62, 95% CI = 0.46 to 0.82; *P* < .001) cancer screening.

An inverse association was found between time since the last medical checkup and cancer screening adherence. Compared with patients with a checkup within the past year, adherence significantly decreased for breast (aOR = 0.58, 95% CI = 0.45 to 0.75; *P* < .001), prostate (aOR = 0.66, 95% CI = 0.47 to 0.91; *P* = .01), and colorectal (aOR = 0.69, 95% CI = 0.56 to 0.86; *P* = .001) cancers when the last checkup was 1 to 2 years prior. For cervical cancer, adherence declined significantly when the last checkup was 2-5 years prior (aOR = 0.26, 95% CI = 0.15 to 0.46; *P* < .001).

Compared with individuals who reported having a personal doctor or a health-care provider, those who answered “No” reported lower odds of USPSTF concordant breast (aOR = 0.68, 95% CI = 0.47 to 0.98; *P* = .04), prostate (aOR = 0.45, 95% CI = 0.31 to 0.65; *P* < .001), and colorectal (aOR = 0.51, 95% CI = 0.40 to 0.66; *P* < .001) cancer screenings.

Furthermore, race was not a significant predictor of screening adherence, with the exception of Black race for breast cancer screening (aOR = 2.09, 95% CI = 1.46 to 3.00; *P* < .001) and Asian American, Native Hawaiian, and Pacific Islander patients for cervical cancer screening (aOR = 0.22, 95% CI = 0.07 to 0.68; *P* = .01).

Table S2 reports the results of the multivariable logistic regression models analyzing predictors of adherence to USPSTF guidelines for breast, cervical, prostate, and colorectal cancer screenings derived from the sensitivity analysis, with individuals reporting solely a history of skin cancer being excluded from this subset. The multivariable model used in the sensitivity analysis found results similar to those obtained from the main analysis model.

## Discussion

This study used nationally representative BRFSS data to examine socioeconomic factors associated with preventive second malignancy screening among cancer survivors for the 4 most common cancers. Prostate cancer screenings showed notably lower adherence to USPSTF guidelines compared with other cancers. Key predictors of reduced screening adherence included low income, lack of health-care coverage, long intervals since the last checkup, and no primary care provider. Our findings highlight inadequate screening among a significant portion of cancer survivors, underscoring the need to address screening disparities and ensure essential preventive care.

Despite recent campaigns to improve cancer screening adherence, particularly for breast cancer, this study reveals that 10%-20% of cancer survivors are not up-to-date on breast, cervical, or colon cancer screenings, and approximately 45% for prostate cancer.^14^ The significant gap in prostate cancer screening rates compared with other cancers aligns with previous studies.^15^ This discrepancy might be attributable to the evolving and sometimes contradictory screening guidelines.^16^^,^^17^ Although adherence to PSA screening for prostate cancer is low, cancer survivors were found to exhibit higher rates than the general population, indicating greater awareness. Considering that we used BRFSS data, these findings may present a more positive view of adherence to USPSTF guidelines than in reality. Considering the increased follow-up needs and focused care required for cancer survivors, these insights hold significant implications.^18^

This study highlights 2 key factors influencing adherence to USPSTF screening guidelines among cancer survivors: The time since the last checkup significantly influenced adherence and the lack of a health-care provider was linked to decreased screening rates for breast, prostate, and colorectal cancers. Consistent medical oversight enhances patient education, promotes health literacy, provides regular screening reminders, and addresses potential screening apprehensions.^19^ This emphasizes the importance of regular medical consultations and informed decision-making with health-care professionals.^20^^,^^21^

Although health-care coverage plays a very large role in access to care—surpassing even the benefits of a substantial household income—the present findings suggest a nuanced picture.^22^^,^^23^ In the primary analysis, health-care coverage was not a significant predictor of USPSTF-concordant screenings, except for colorectal cancer. However, when excluding individuals with only skin cancer, coverage was an independent predictor of adherence to breast, prostate, and colorectal screenings. Past studies suggest limited coverage hinders access to screenings and is linked to later-stage diagnoses.^24^ Although the Affordable Care Act reduced the uninsured population and eliminated in-network cost-sharing for some preventive services, disparities in cancer screening adherence persist.^25-27^ These findings highlight the need for targeted strategies beyond insurance provision to bridge these preventive care gaps.^28^

Our findings indicate that lower annual household income consistently correlated with decreased odds of screening adherence across all cancer types in both analyses. The economic burden postcancer diagnosis, including income, is well documented in the literature.^22^^,^^29^^,^^30^ Patients may deplete significant savings, accrue debt, or leverage vital assets.^31^ This might lead to reduction in medical visits, medication underprescription, and expense cutbacks,^22^^,^^23^ deterring adherence to cancer screenings, especially among the uninsured. These pressures may also exacerbate mental health issues such as anxiety and depression.^32^^,^^33^

Race/ethnicity variations in adherence to USPSTF cancer screenings were observed, but inconsistently across cancer types. No single racial/ethnic group consistently showed lower adherence odds, suggesting that other factors may influence adherence more among cancer survivors than in the general population.^34^

Our study is one of the first to evaluate cancer screening adherence among survivors, with a unique focus on socioeconomic factors. Moreover, by using contemporary data, we enhance the generalizability of our findings within the current US cancer care landscape. Previous data from the 2021 National Health Interview Survey (NHIS) found adherence rates of 75.9% for breast, 72.4% for cervical, 37.1% for prostate, and 71.8% for colorectal cancer screening among the general population without a prior cancer diagnosis.^35^ In our study, cancer survivors were found to exhibit a heightened awareness of screening importance, with USPSTF-concordant adherence rates surpassing those of the general population for all examined cancers. However, comparisons between BRFFSS and NHIS data may be challenging. Several factors likely contribute to the differences in screening estimates between BRFSS and NHIS, including nonresponse bias, because BRFSS has lower response rates. Additionally, differences in question order, weighting procedures, sample design, and survey modality may influence the comparison of their estimates.^15^

### Limitations

This study presents some limitations. BRFSS data, relying on self-reported data, may be inaccurate due to memory lapses or misrepresentation. Telephone interviews may exclude those without telecommunication access or language proficiency. Additionally, the 47.9% national response rate raises concerns about generalizability. Excluding individuals with incomplete data on prior cancer or screening history leads to selection bias, potentially compromising the generalizability of findings on screening adherence. Moreover, a high missing information rate on cancer types introduces further bias, because participants might be misclassified for screenings despite prior diagnoses of the same cancer. Additionally, the dataset lacks information on the timing of cancer diagnoses, introducing another potential selection bias, because some screenings may have occurred before diagnosis, which may not fully represent the screening experience of cancer survivors. Another limitation to consider with BRFSS data is the potential overestimation of cancer screening rates, likely influenced by factors such as nonresponse bias.^15^ Finally, the analyses were conducted using 2020 BRFSS data, with reported screening rates likely affected by the COVID-19 pandemic. For instance, a modest decrease in breast and cervical cancer screenings was reported in recent studies.^36^

## Conclusions

Despite their elevated risk, cancer survivors show disparities in adherence to USPSTF screening guidelines. Lower income, lack of health-care coverage, absence of dedicated providers, and long intervals since the last checkup are key factors. Racial or ethnic backgrounds did not consistently influence these behaviors. Targeted interventions accounting for such factors may help mitigate these disparities.

## Supplementary Material

pkae127_Supplementary_Data

## Data Availability

The data used in this study were derived from sources in the public domain: the 2020 Behavioral Risk Factor Surveillance System survey (https://www.cdc.gov/brfss/annual_data/annual_2020.html).
